# Nuclear envelope components in vascular mechanotransduction: emerging roles in vascular health and disease

**DOI:** 10.1080/19491034.2025.2453752

**Published:** 2025-01-19

**Authors:** Tung D. Nguyen, Michael A. Winek, Mihir K. Rao, Shaiva P. Dhyani, Monica Y. Lee

**Affiliations:** a Department of Physiology and Biophysics, The University of Illinois at Chicago – College of Medicine, Chicago, IL, USA; b The Center for Cardiovascular Research, The University of Illinois at Chicago – College of Medicine, Chicago, IL, USA

**Keywords:** Cardiovascular disease, endothelial cells, LINC complex, nuclear lamina, nuclear pore complex, vascular smooth muscle cells

## Abstract

The vascular network, uniquely sensitive to mechanical changes, translates biophysical forces into biochemical signals for vessel function. This process relies on the cell's architectural integrity, enabling uniform responses to physical stimuli. Recently, the nuclear envelope (NE) has emerged as a key regulator of vascular cell function. Studies implicate nucleoskeletal elements (*e.g.* nuclear lamina) and the linker of nucleoskeleton and cytoskeleton (LINC) complex in force transmission, emphasizing nucleo-cytoskeletal communication in mechanotransduction. The nuclear pore complex (NPC) and its component proteins (*i.e.* nucleoporins) also play roles in cardiovascular disease (CVD) progression. We herein summarize evidence on the roles of nuclear lamina proteins, LINC complex members, and nucleoporins in endothelial and vascular cell mechanotransduction. Numerous studies attribute NE components in cytoskeletal-related cellular behaviors to insinuate dysregulation of nucleocytoskeletal feedback and nucleocytoplasmic transport as a mechanism of endothelial and vascular dysfunction, and hence implications for aging and vascular pathophysiology.

## Introduction

**Figure 1.**
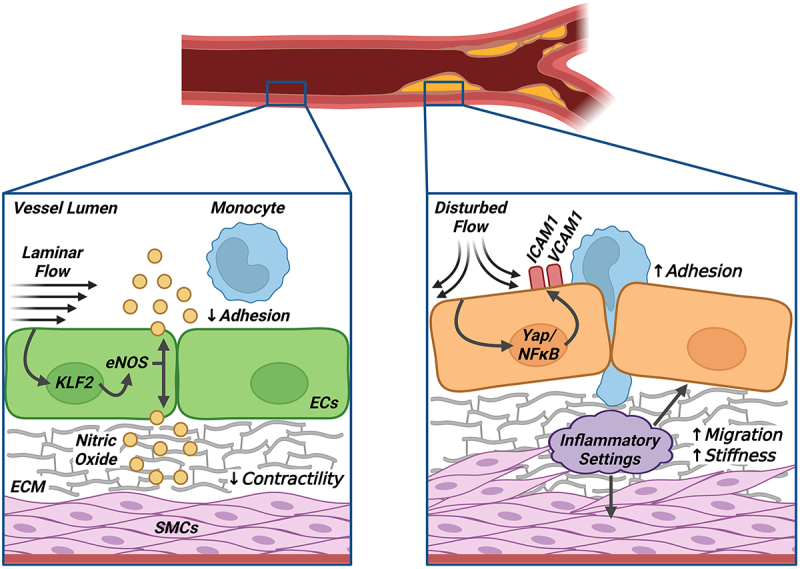
The vascular endothelium serves as a selective monolayer for vessel health. (a) Endothelial cells (ECs) serve as the innermost layer of the vasculature, functioning as a dynamic and selective barrier between circulating blood components and surrounding tissue. In response to laminar flow, ECs upregulate endothelial nitric oxide synthase (eNOS) expression for subsequent nitric oxide (NO) production. As a gaseous cardioprotective agent, NO reduces ec-leukocyte interactions and promotes vasodilatory effects through its action on vascular smooth muscle. Laminar flow also prompts cytoskeletal remodeling and EC alignment along the direction of flow, a well-known mechano-driven mechanism of EC protection. (b) Disturbed flow conditions, on the other hand, promote EC transition toward an inflammatory state. EC dysfunction is triggered by the nuclear translocation of several transcriptional activators (*e.g*. Yap, nf-kB), leading to increased adhesion molecule expression (ICAM-1, VCAM-1) and monocyte extravasation. EC dysfunction additionally leads to barrier leakage, medial layer stiffening, and SMC phenotypic switching, thereby leading to the pathogenesis of cardiovascular diseases.

The adult vasculature is one of the largest organ systems in the human body. Broadly classified into arteries, veins, or capillaries, a healthy vasculature is required for proper blood circulation and nutrient delivery to peripheral tissue [1,2]. Arteries, in comparison to other vessel types, have revealed unique physiological properties to precipitate the pathogenesis of CVD, such as atherosclerosis (Figure 1). The arterial wall is composed of three distinct layers, namely the tunica adventitia, tunica media, and tunica intima. The tunica adventitia forms the outermost layer of the vasculature and provides structural support via fibroblasts and other stromal tissues [3]. The middle layer, or tunica media, consists mainly of vascular smooth muscle cells (SMCs) and various extracellular matrix (ECM) proteins (*e.g*. collagen, fibronectin) that influence vessel reactivity [4–6]. The tunica intima, or innermost layer of the vasculature, serves as a dynamic barrier between circulating blood components and tissue. As such, this layer is continuously exposed to various blood flow patterns and extracellular stimuli, rendering it highly susceptible to inflammation, an underlying driver for vascular disease and atheroma progression [7,8].

The tunica intima is composed of a continuous monolayer of quiescent, yet fully responsive endothelial cells (ECs) [9,10]. This low-permeability, single-cell lining actively regulates the movement of solutes and macromolecules while also maintaining a non-thrombogenic surface for unobstructed blood flow [11,12]. EC-mediated regulation of vessel permeability is largely attributed to the opening and closing of cell-cell junctions. The disassembly and reassembly of both adherens (*e.g*. VE-Cadherin) and tight junction (*e.g*. Claudin, Occludin) proteins coordinates paracellular permeability to therefore control the passage of solutes and proteins into residing tissue [13–15]. Leaky endothelial junctions lead to features of vascular dysfunction, including increased vessel inflammation and impaired stimuli-induced vasodilation. Maintaining endothelial barrier integrity is therefore essential for vascular health and homeostasis.

As the only vascular cell type exposed to hemodynamic stress, ECs are uniquely sensitive to fluid mechanical forces. Endothelial exposure to a unidirectional, laminar flow profile induces both biochemical and morphological changes, eliciting an atheroprotective endothelial phenotype. Decades of work establish Kruppel-like factor (KLF)-mediated upregulation of endothelial nitric oxide synthase (eNOS) and subsequent nitric oxide (NO) production as a major mechanism of laminar flow-induced endothelial protection (Figure 1a) [16,17]. As one of the most cardioprotective agents produced by ECs, NO is an indispensable molecule involved in anti-inflammation to include various protective mechanisms, such as decreased EC-leukocyte adhesion through inhibition of inflammatory cell adhesion molecules (*e.g*. ICAM-1, VCAM-1) [16,18]. Conversely, a disturbed and turbulent blood flow pattern triggers EC activation, or the phenotypic conversion from a quiescent to a proinflammatory, atherogenic state. This involves the increased nuclear enrichment of inflammatory transcription factors, including Yes-associated protein (Yap) and Nuclear factor-κB (NFκB), thus promoting the progression of chronic vascular diseases (*e.g*. atherosclerosis) (Figure 1b) [19–21]. Endothelial activation is further characterized by a decline in NO production and bioavailability, provoking a multitude of vascular complications associated with disease, including impaired vascular SMC relaxation and arterial stiffening [22,23]. Understanding the differential impact between laminar and turbulent flow profiles on EC health and function is thus critical for developing therapeutic strategies to combat vascular disease progression.

**Figure 2.**
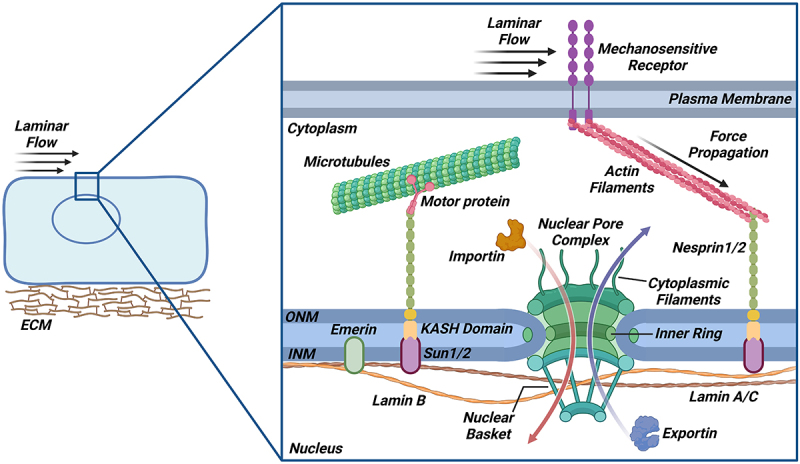
Nuclear envelope components regulate various features of vascular cell biology. The nuclear lamina fibers, comprised of lamin A/C and Lamin B, line the inside of the nuclear membrane to confer nuclear structural stability, providing a binding platform for nuclear proteins and chromatin. The linker of nucleoskeleton and cytoskeleton (LINC) complex physically couples both actin filaments and microtubules (via motor proteins) to the nucleus and consists of kash-domain nesprin proteins in the outer nuclear membrane (ONM) and Sun domain proteins in the inner nuclear membrane (INM). Mechanical forces stemming from physiological laminar flow are recognized by mechanosensitive receptors, which propagate these signals toward the nucleus via the cytoskeleton and LINC complex proteins. The nuclear pore complex (NPC) regulates the selective nucleocytoplasmic exchange of biomolecules by permitting the rapid diffusion of small molecules while requiring larger molecules to utilize an active transport system. Cytoplasmic filaments and nuclear baskets allow for docking of larger cargos before they are shuttled through the NPC central channel via interactions between nuclear transport proteins (*i.e*. Importin, Exportin) and the inner ring proteins.

The majority of endothelial mechanotransduction studies have focused on plasma membrane-localized components for the sensing of fluid shear stress [24,25]. Recent evidence, however, suggests that NE components are also critical mediators of endothelial health and flow adaptation [26–28]. Much like the plasma membrane, the NE not only functions to establish compartmentalization, but also as a highly dynamic force sensor [29]. The NE is comprised of two lipid bilayers, defined as the outer nuclear membrane (ONM) and the inner nuclear membrane (INM) (Figure 2). Within the NE spans the LINC complex, a structure that physically connects the cytoskeletal network to the nucleus to create a mechanically coupled unit [29,30]. Recent studies report that disruption of nucleoskeletal (*e.g*. lamins [28] and nuclear-cytoskeletal connections (*e.g*. LINC proteins [27] prevents EC flow adaptation, leading to increased inflammation and permeability [31–33]. Hence, the nucleus is physically tethered to the necessary mechanosensory elements to both perceive and transmit mechanical cues from the extracellular environment. The nucleus itself has also been reported as a direct sensor of blood flow in ECs, as endothelial nuclei are displaced by hydrodynamic drag to thus affect EC polarization under flow [34]. Understanding the role of NE components in EC biology has become an emerging interest, considering EC flow responsiveness and mechanotransduction relies on actin filament remodeling to affect whole-cell structural integrity.

### Lamina fibers confer nuclear structural stability

The structural integrity of the nucleus is a determining factor in the cellular ability to withstand mechanical stress [35]. A major structural element of the NE includes the nuclear lamina, a dense meshwork of proteins that line the INM (Figure 2) [36–38]. Classified as type V intermediate filaments, lamin proteins contain the typical domain structure of the intermediate filament superfamily, which includes an N-terminal head domain, a coiled-coil central α-helical rod domain, and a C-terminal immunoglobulin-like tail domain [36,38]. The nuclear lamina is composed of A-type and B-type lamins to give rise to four lamin isoforms. A-type lamins are expressed by the *LMNA* gene, where the most prominent post-transcriptional splicing events lead to Lamin A and Lamin C proteins [38,39]. B-type lamins are divided into Lamin B1 and Lamin B2 proteins that are expressed by the *LMNB1* and *LMNB2* genes, respectively [38,39]. These proteins are abundant and ubiquitously expressed in most differentiated somatic cells, including cardiovascular cells [26,38,40,41].

To form the characteristic fibrous meshwork found on the INM surface, the lamin proteins interact to form dimers that polymerize into thick filaments and thus confer structural support for a properly shaped nucleus [39]. *In vitro*, both A-type and B-type lamins form coiled heterodimers as well as homodimers of only one type of lamin protein [39]. A-type lamins are found to play key roles in maintaining nuclear shape and membrane integrity, critical for cells under mechanical stress [42]. Consequently, fibroblasts isolated from LMNA-deficient mice showed substantial nuclear deformity and softening for impaired cell viability, suggesting that homodimeric A-type lamins may help stabilize the NE for overall cellular health (Table 1) [32,43]. On the other hand, B-type lamins have been associated with cellular senescence and DNA replication [70]. Nonetheless, both A- and B-type lamins, have recently been shown to interact with nucleoporin proteins (*e.g*. Nucleoporin153 [Nup153]) to anchor and regulate the positioning of nuclear pores in various cell types [36,38]. Moreover, both lamin types perceive mechanical force propagation from the cell surface through their physical coupling with LINC complex components [39]. Lastly, both lamin types have been shown to interact with histones and other lamina-associated proteins to influence chromatin interactions at the nuclear periphery, further implicating the nuclear lamina in mechanosignaling and gene regulation [56,57].

**Table 1.** A table listing nuclear envelope-associated proteins and relevant studies across different vascular cell types. The table includes protein names, associated cell types, and key findings from each study.

NE Component	Protein	Cell Type/ Organism	Experimental Observation	References	
Nuclear Lamina	Lamin A/C	MEF	Depletion leads to severe nuclear deformity and softening for impaired NF-κB-mediated anti-apoptotic signaling and enhanced cell apoptosis in response to mechanical stress.	[32,43]	Lammerding, J. et al., 2004; Kolb, T. et al., 2011
		SMC	Accumulation of truncated mutants (progerin) at the NE precipitates nuclear stiffness for sustained DNA damage and premature aging phenotypes (VSMC death, vessel fibrosis, advanced plaque formation).	[44–46]	Scaffidi, P. & Misteli, T., 2006; Hamczyk, M. R. et al., 2018; Coll-Bonfill, N. et al., 2023
		MAEC	Progerin expression leads to increased actin stress fiber formation and EC misalignment under laminar flow for loss of eNOS expression and decreased NO production.	[28,47]	Osmanagic-Myers, S. et al., 2018; Fang, F. et al., 2011
		HCAEC	Progerin expression results in sustained oxidative DNA damage, impaired DNA repair mechanisms, and premature cell senescence for enhanced EC inflammation and eNOS loss. Depletion leads to cytoskeletal softening and deformed cell nuclei for enhanced subendothelial trafficking of T cells and monocytes.	[48,49]	Gordon, L. B. et al., 2016; Bidault, G. et al., 2020
	Lamin B1	Fibroblast	Mutations selectively impairs Nup153 localization to the NE for elevated Nup153 enrichment in the nucleoplasm (laminopathy patient-derived).	[36,38,50]	Hutchison, C. J., 2002; Ho, C. Y. & Lammerding, J., 2012; Al-Haboubi, T. et al., 2011
		MCF7 U2OS	Depletion results in cytoskeletal clumping of LINC complex proteins for elevated nuclear deformity and premature cell senescence.	[51]	Lammerding, J. et al., 2005
		C. elegans	Co-depletion with Lamin A/C leads to abnormal clustering of NPCs toward one side of the cell nuclei.	[52]	Liu, J. et al., 2000
LINC Complex	KASH	SMC	Expression of exogenous KASH2 attenuates nuclear deformities and reduces the expression of cell senescence markers under cyclic strain to restore medial VSMC density and reduce vessel fibrosis.	[53]	Kim, P. H. et al., 2018
		HUVEC	Expression of exogenous DN-KASH impairs cell-cell and cell-matrix adhesions at rest and under cyclic strain as well as prevents EC realignment under laminar flow for impaired tube formation.	[27,54,55]	Denis, K. B. et al., 2021; Lombardi, M. L. et al., 2011; Lityagina, O. & Dobreva, G., 2021
	Nesprin-1/ Nesprin-2	HUVEC	Co-depletion leads to severe nuclear deformities and impairs actin cytoskeletal reorientation for diminished cell migration and tube formation. Nesprin-1 depletion alone reduces focal adhesion turnover to impair EC realignment and migration under cyclic strain.	[56–58]	Gruenbaum, Y. et al., 2005; Uhler, C. & Shivashankar, G. V., 2017; Muchir, A. et al., 2007
		RAEC	Nesprin-2 loss under low shear stress increases the phosphorylation and nuclear localization of transcription factors involved in EC proliferation and apoptosis.	[59,60]	Anno, T., Sakamoto, N. & Sato, M., 2012; Han, Y. et al., 2015
	Sun1	SMC	Co-depletion with Sun2 leads to impaired RhoA signaling kinetics and altered actomyosin-induced cell spreading.	[61]	Porter, L. et al., 2020
		HUVEC	Depletion results in destabilized cell-cell junctions and impaired EC junctional realignment under laminar flow as well as enhances RhoA activity for increased actin stress fiber formation and EC hyper-contractility.	[62]	Buglak, D. B. et al., 2023
		MEF HeLa	Depletion leads to abnormal aggregation of NPCs at random clusters in the nuclear membrane.	[46]	Liu, Q. et al., 2007
	Sun2	SMC	Depletion with treatment of actomyosin activity inhibitor impairs mobile traction along the inner nuclear membrane.	[61]	Porter, L. et al., 2020
Nuclear Pore Complex	POM121	U2OS	Co-depletion with Sun1 results in perturbed NPC insertion across the NE during interphase and impairs the formation of new NPCs.	[63]	Talamas, J. A. & Hetzer, M. W., 2011
	Nup93	CM	Aberrant expression alongside Nup153, Nup160, and NDC1 correlated with worsened ventricular function (cardiomyopathy patient-derived).	[64,65]	Burdine, R. D. et al., 2020; Tarazón, E. et al., 2012
		HREC	Depletion leads to nuclear leakiness and Yap nuclear accumulation for EC senescence and downstream endothelial inflammation (increased adhesion molecule expression, enhanced EC-monocyte interactions).	[66]	Nguyen, T. D. et al., 2024
	Nup62	CM	Depletion results in increased cell nuclei size, impaired localization of nuclear transport proteins, and perturbed nuclear trafficking of NLS-tagged exogenous constructs.	[67]	Chahine, M. N. et al., 2015
	Nup155	CM	Depletion or phosphodeficient mutation leads to altered nuclear permeability and inhibits nuclear import of Hsp70 for diminished action potential duration and progression of heart failure.	[68]	Zhang, X. et al., 2008
	Nup153	HUVEC	Depletion or pharmacological inhibition by OMe-Syn impairs growth factor-induced angiogenesis and cell viability.	[69]	Kim, N. H. et al., 2015

MEF: mouse embryonic fibroblast; VSMC: vascular smooth muscle cell; MAEC: mouse aortic endothelial cell; HCAEC: human coronary artery endothelial cell; HUVEC: human umbilical vein endothelial cell; RAEC: rat aortic endothelial cell; HREC: human retinal endothelial cell, CM: cardiomyocyte, NLS: nuclear localization sequence.

Recent work has also identified a role for endothelial lamins in immune cell behavior. Both the innate (*e.g*. monocytes, macrophages) and adaptive (*e.g*. T and B cells) immune system play key roles in atherogenesis. Subendothelial retention of lipoproteins and various pro-inflammatory factors trigger endothelial activation for the expression of adhesion molecules that mediate the attachment of circulating monocytes and other leukocytes. Both monocytes (*i.e*. pre-differentiated macrophages) and T cells utilize intraluminal crawling and subsequent trans-endothelial migration to infiltrate the subendothelial space [71,72]. Prior to breaching the EC barrier, monocytes and T cells have been demonstrated to preferentially avoid crawling over stiff endothelial nuclei in cell culture experiments [41]. In these studies, knockdown of endothelial Lamin A results in a softer cytoskeletal structure and deformed cell nuclei, allowing both immune cell types to make prolonged contact with the apical surface of Lamin A-deficient ECs for enhanced subendothelial trafficking (Table 1) [41,73]. In other words, endothelial loss of Lamin A, and hence a decrease in nuclear stiffness, leads to more frequent T cell crawling, suggesting that stiffer endothelial nuclei can act as a repulsive cue to minimize interactions between ECs and immune cells. As such, this work identifies endothelial lamin A, and nuclei stiffness, as a negative regulator of EC-leukocyte interaction. Given the multicellular interplay in atherosclerotic lesion development, it is intriguing to observe reports of immune cell transmigration regulation at the level of the endothelial nuclei, highlighting previously unrecognized susceptibilities in CVD development [41,73].

### Progerin laminopathies in vascular cell types

Aberrations in Lamin A expression have been well-characterized, with over 400 distinct mutations of the *LMNA* gene linked to various mammalian diseases, termed laminopathies [58,74]. Critical for genomic stability and whole-cell mechanics, several laminopathies manifest across a wide range of human diseases broadly defined as ‘premature aging disorders’, including Hutchinson-Gilford Progeria Syndrome (HGPS). HGPS is a rare, fatal, and genetic condition in which children display striking phenotypes resembling that of premature aging. Some laminopathies also promote disease in selective organs yet the underlying mechanisms conferring tissue-specific pathology remains elusive. With roles in nuclear structural support and gene expression, the tissue-selective nature of laminopathies may reflect an outcome of mechanical damage and/or pathogenic changes in gene expression [36].

With the exception of Lamin C, the C-terminal domain of mammalian lamins contains a cysteine-aliphatic-aliphatic-any amino acid (CAAX) motif that triggers a series of enzymatic reactions to generate mature lamin proteins. First, a prenyl group (*i.e*. farnesyl moiety) is added to the cysteine residue of the C-terminus CAAX motif by protein farnesyltransferase (FTase). Second, an endoprotease specific for prenylated proteins cleaves off the last three amino acids (−AAX). Third, the newly exposed farnesylcysteine is methylated by isoprenylcysteine carboxyl methyltransferase (ICMT). Lastly, the farnesylated protein is recognized by Zinc Metallopeptidase STE24 (ZMPSTE24) for cleavage of the last 15 amino acids, including the farnesylcysteine α-methyl ester, to generate mature unfarnesylated and unmethylated lamin protein [75]. HGPS is caused by a *de novo* point-mutation in LMNA that results in an in-frame deletion of 50 amino acids to impair normal LMNA processing, resulting in a mutant lamin A protein, also known as progerin. While prelamin A is only temporarily tethered to the nuclear membrane through farnesylation and ultimately incorporated into the lamina meshwork upon maturation, pre-progerin is irreversibly farnesylated and thus remains permanently anchored to the nuclear membrane [44,48]. Aggregation of the progerin protein at the nuclear membrane is heavily implicated in the pathogenesis of HGPS, where patients typically face adolescent mortality from complications associated with occlusive coronary and/or cerebrovascular disease [76–81]. Atherosclerotic lesions isolated from genetically-confirmed cases of HGPS display heightened progerin localization at the nuclear membrane in vascular SMCs within severe plaque regions [80]. A hypercholesterolemic, atherosclerosis-prone murine model of HGPS (*i.e. ApoE*^*–/–*^*/LMNA*^*LCS/LCS*^*/SM22α-Cre*) was also reported to develop substantial vascular pathologies, including loss of medial vascular SMCs, thickening of the adventitial layer, and accelerated atherosclerotic plaque development [45]. The accumulation of progerin in vascular cell types (*i.e*. SMCs, fibroblasts), as part of HGPS disease progression, inadvertently impairs nuclear stiffness, leads to dysmorphic cell nuclei, and increases DNA damage (Table 1) [44–46]. Progerin-afflicted vascular cells also exhibit a delay in the recruitment of DNA repair proteins, further exacerbating DNA damage to induce premature cellular senescence [82].

A prominent vascular feature of HGPS includes loss of SMCs in the large arteries [45,83]. A recent study attributes HGPS-associated vascular SMC loss to phenotypic switching, where progerin-expressing SMCs alter cellular identity toward a ‘synthetic’ state, exhibiting decreased contractile protein expression, increased replicative stress, and genomic instability [46]. Intriguingly, SMC-specific progerin expression mirrors the vascular abnormalities described in human HGPS, suggesting that the premature aging phenotypes and vascular complications largely reflect intrinsic defects of progerin-expressing SMCs [45]. The cellular heterogeneity of the vasculature, however, complicates our understanding of HGPS disease mechanisms. While vascular abnormalities in HGPS have largely been attributed to progerin accumulation in SMCs, recent reports implicate progerin expression in ECs as an additional mechanism of HGPS-related vascular dysfunction. Similar to vascular SMCs, endothelial progerin expression impairs EC responses to mechanical stimuli to include abnormal vessel reactivity [28,49]. Endothelial-specific expression of progerin was reported to increase actin stress fiber formation and impair endothelial alignment in vessel regions exposed to a unidirectional laminar flow profile (*e.g*. thoracic aorta) (Table 1) [28]. Mechanistically, progerin-expressing ECs exhibit an accumulation of myocardin-related transcription factor-A (MRTF-A) at the nuclear periphery, a known repressor of the eNOS promoter. As such, progerin-expressing ECs express significantly less eNOS protein and NO levels to precipitate pro-inflammatory and pro-fibrotic vascular phenotypes, such as myocardial thickening and perivascular inflammation [28,47]. Exogenous progerin expression in cultured human ECs induced similar observations of accumulated DNA damage as with progerin buildup in vascular SMCs, including oxidative DNA damage, impaired DNA repair mechanisms, and premature cellular senescence [44,49]. Demonstrating the vital importance of vascular health, progerin expression in both ECs and SMCs gives rise to a combination of intrinsic and extrinsic consequences, resulting in various vascular complications.

### LINCing the cytoskeleton to the nuclear lamina

The cellular cytoskeleton is physically and functionally coupled to the nucleoskeleton through INM and ONM-localized proteins, also referred to as the LINC complex. In mammals, the Sad1p/UNC-84 (SUN) protein family includes five isoforms which reside at the INM. SUN1 and SUN2 are ubiquitously expressed and implicated in several cardiovascular cell types, including cardiomyocytes, vascular SMCs, and ECs [84]. SUN2 contains a single transmembrane domain spanning the INM, whereas SUN1 possesses three [59]. The N-terminal domain of SUN1 interacts with both the nuclear lamina (*e.g*. Lamin A/C) and nuclear pore complex (NPC) proteins (*i.e*. nucleoporins) to suggest a role in NE architecture and/or spatial regulation of INM proteins [85]. Recent findings also highlight the differential influences of nuclear lamin isoforms (*i.e*. A-type vs. B-type) on LINC complex-dependent nucleocytoskeletal coupling [86]. Although both A-type and B-type lamins regulate cytoplasmic stiffness and contractility through LINC-mediated interaction with vimentin intermediate filaments, only A-type lamins engage with LINC-actin filaments to influence cortical stiffness [86]. Further demonstrating the differential influences of isoform types, SUN1 engages with microtubules whereas SUN2 interacts with actin for nuclear positioning [87]. The highly-conserved C-terminal domain of SUN1/SUN2 extends into the perinuclear space to interact with ONM-residing Nesprin proteins (*i.e*. Nesprin-1/2) [88,89]. In humans, four nesprin encoding genes (*SYNE)* have been identified to date. Nesprin-1 and Nesprin-2 are the largest isoforms and contain an N-terminal actin-binding domain for interaction with cytoplasmic actin filaments [61,90]. Nesprin3 exists as two isoforms (Nesprin-3α and Nesprin-3β), with Nesprin-3α as the predominant isoform reported to bind intermediate filaments via plectin [62]. Nesprin4, primarily expressed in secretory epithelial cells, is a kinesin 1-binding protein that connects the nucleus to microtubules [91]. The Nesprin proteins contain a Klarsicht/ANC-1/Syne homology (KASH) transmembrane domain at the C-terminus that is essential for localizing proteins to the ONM and interacting with SUN domain proteins. Altogether, a connective bridge across the perinuclear space, formed between SUN proteins (*i.e*. SUN1/SUN2) and the KASH domain of Nesprin proteins, effectively connects the nuclear lamina to the cytoskeleton (Figure 2) [88,89]. The LINC complex hence confers structural support while is also receptive to intracellular force transmission to play a major role in cytoskeletal-dependent cellular processes, such as cell polarization and orientation [54].

ECs are highly responsive to external mechanical stimuli and susceptible to changes in fluid shear stress and cyclic biaxial stretching [92]. These external cues, recognized by various mechanosensitive receptors, are transduced into signals that propagate information toward the NE, relying on a network of actin and other cytoskeletal filaments (Figure 2) [88,93]. Stable interactions between the KASH (Nesprin)-SUN bridges are seemingly necessary to transfer the mechanical forces across the double membrane NE to exert downstream effects. As such, recent studies have implicated LINC complex components in transmitting cyclic stretch and shear flow-associated mechanical forces in ECs [60,88,93]. Furthermore, INM-located Emerin proteins have also been shown to restructure nuclear lamina fibers and alter the chromatin landscape in response to environmental forces [30,94]. The physical tethering of cytoskeletal components to the nucleus is therefore essential for whole-cell mechanotransduction and cellular function at various levels of regulation.

### The LINC complex in cardiovascular disease

As major INM components of the LINC complex, SUN proteins physically couple with Nesprin proteins to connect the nuclear lamina meshwork with cytoskeletal filaments (*e.g*. actin filaments, microtubules). This nuclear-cytoplasmic tethering is necessary for proper cell morphology and adherence to neighboring cells and ECM products [95]. Similar to lamins, aberrations in LINC proteins (*e.g*. SUN1/2, Emerin) have also been associated with disease and features of premature aging [51,96,97]. Implicating a role for SUN1 in aging, SUN1 overaccumulation is a common finding in mouse models of laminopathies and premature aging (*i.e. LMNA*^*-/-*^, *LMNAΔ9)* [98]. Fibroblasts from HGPS patients and naturally aged individuals also exhibit increased SUN1 protein levels and a consequent increase in nuclei-associated microtubules [96]. This imbalance in nucleocytoskeletal connections may explain the cell polarity defects associated with physiological aging. Emerging studies also identify SUN proteins as major regulators of actomyosin activity and cytoskeletal reorganization in vascular cells; loss of SUN proteins in human SMCs impairs RhoA signaling kinetics for defective actomyosin-mediated SMC spreading (Table 1) [99]. Intriguingly, pharmacological inhibition of actomyosin activity in SMCs selectively reduces SUN2, and not SUN1, interaction with Lamin A and leads to decreased SUN2 mobility in the INM. These findings suggest that actomyosin-generated forces engage LINC complex tension via increased SUN2-Lamin A interaction as a mechanism of biomechanical feedback [99].

SUN proteins have also recently been demonstrated to play a key role in maintaining endothelial health. Knockdown of endothelial SUN1 leads to decreased trans-endothelial electrical resistance, a readout of destabilized cell-cell junctions, and impaired EC alignment under physiological laminar flow exposure [100]. Loss of SUN1 also enhanced actin stress fiber formation and actomyosin-induced EC hypercontractility, and pharmacological inhibition of actomyosin activity was sufficient to restore EC rigidity and junctional integrity [100]. The increase in actin stress fiber formation and EC permeability upon SUN1 knockdown was later attributed to elevated RhoA activity [100]. RhoA signaling can be activated by the microtubule-associated RhoGEF-H1, where associated RhoGEF-H1 protein remains in an inactive state when bound to microtubules [101]. Mechanistically, loss of endothelial SUN1 was found to destabilize peripheral microtubules and hence increase free, cytosolic RhoGEF-H1, thereby increasing RhoA activity for consequent EC permeability. As such, RhoGEF-H1 depletion rescued the effects of SUN1 knockdown. Similarly, Nesprin-1 knockdown in SUN1-deficient ECs restored endothelial barrier function, indicating that Nesprin-1 is necessary for communicating SUN1-mediated effects on EC junctions to implicate LINC proteins in EC behavior.

Receptive to external forces, LINC complexes also integrate signals from focal adhesions [27,102]. Serving as mechanical links between the intracellular actin network and the ECM, focal adhesions play a key role in endothelial sensing of the biophysical environment [103]. These dynamic macromolecular assemblies are sensitive to shear flow patterns, capable of discerning disturbed flow patterns for integrin activation and EC inflammation [104]. The disassembly of focal adhesions is a necessary process for cellular migration and reorientation. The Nesprin proteins (*e.g*. Nesprin-1/2) bind to actin filaments, and aberrant Nesprin expression has been shown to precipitate severe defects in EC morphology and function (Table 1) [27,31,33,60]. For example, endothelial knockdown of Nesprin-1 and Nesprin-2 has been reported to increase focal adhesion number, enhance traction forces, and decrease migration speed [31,33]. Nesprin-1-deficient ECs also exhibit abnormal cellular orientation in response to cyclic strain, suggesting a role for Nesprin-1 in nuclear-cytoskeletal connection and mechanical communication [33]. Nesprin-2 protein levels are also substantially reduced in ECs exposed to atheroprone low shear stress (5 dynes/cm^2^), a flow pattern predominantly found at arterial bifurcations and other lesion-prone curvatures, in comparison to ECs exposed to healthy physiological shear stress (15 dynes/cm^2^) [105]. This decrease in Nesprin-2 levels also resulted in the increased phosphorylation and nuclear localization of Activator protein-2 (AP-2) and Transcription factor IID (TFIID), transcription factors involved in EC proliferation and apoptosis. Intriguingly, restoring Nesprin-2 expression in ECs exposed to low shear flow was sufficient to attenuate active AP-2 and TFIID levels and the consequent proliferation- and apoptosis-associated transcriptional profile [105], demonstrating the importance of LINC proteins in EC function.

Recent translational work has focused on the potential role of Nesprin proteins, namely their KASH domains, in restoring progerin-afflicted SMC health. Several studies indicate an increase in nuclei stiffness with progerin expression, suggesting that progerin toxicity could be mitigated by reducing force transmission to the nucleus [106–108]. Expression of the KASH domain promiscuously binds to endogenous SUN proteins, thereby displacing endogenous Nesprins from the NE to effectively disrupt physical coupling between the cytoskeleton and the nuclear lamina [53,55,109]. Interestingly, exogenous KASH expression was reported to attenuate nuclear deformations and concurrently reduce levels of cell senescence markers (*e.g*. p53, γH2AX) in progerin-expressing SMCs under cyclic strain. Furthermore, KASH expression driven by an SM22α-Cre transgene (*i.e*. SMC-specific) restored medial SMC density and ameliorated adventitial fibrosis in both the thoracic aorta and the aortic arch of HGPS mice to levels comparable to that of wild type mice [53]. These findings suggest that expression of exogenous KASH effectively disrupts force propagation toward the nuclei, thus limiting the detrimental effects of progerin (Table 1) [53].

Expression of a KASH domain construct interferes with endogenous SUN-Nesprin interactions to disrupt nucleo-cytoskeletal connections, and can therefore serve as a valuable tool to study nucleocytoskeletal coupling [27,54,55]. To detail the underlying role of the LINC complex in EC behaviors, several groups have utilized KASH expression constructs (*i.e*. DN-KASH) to disrupt the LINC complex in non-HGPS ECs. Human ECs expressing DN-KASH was reported to impair EC barrier function; DN-KASH expressing ECs exhibit a significant reduction in cellular impedance and a corresponding increase in paracellular pore formation (Table 1) [27]. Exogenous DN-KASH expression also led to notable defects in endothelial cell-cell and cell-ECM adhesion, both at rest and under cyclic stretching, suggesting that nucleocytoskeletal coupling is required for EC adhesion and mechanical stress adaptation. Likewise, endothelial DN-KASH expression impaired endothelial alignment upon exposure to physiological laminar flow (15 dynes/cm [2,27]. This was paralleled by an increase in endothelial detachment and impaired angiogenic paradigms (*e.g*. migration, sprouting) to indicate focal adhesion-dependent processes also rely on intact LINC complexes. SUN-Nesprin interactions are clearly necessary for various EC functions, giving rise to the importance of nucleocytoskeletal force transmission in mechanical stability. Disrupting LINC complexes is, nonetheless, dependent on cell type- and state, as exogenous KASH expression was again revealed to exert therapeutic effects to attenuate progerin-mediated vascular pathology [53]. These recent studies begin to expose the long-range biophysical communication capacity within vascular cells, where defects at the level of the NE can manifest toward whole-cell dysfunction and devastating physiological consequences.

### NPCs regulate nucleocytoplasmic exchange

The physical separation of genomic material from the cytoplasm in eukaryotes is a notable example of how compartmentalization provides organization in complex biological systems. The controlled exchange of molecules between the nucleus and cytoplasm occurs through NPCs, one of the largest macromolecular complexes (~125MDa) of the NE [63,64]. The human NPC is a highly organized unit composed of ~ 30 different protein subunits known as nucleoporins, which form a nucleocytoplasmic gateway [110]. These 8-fold rotationally symmetric NPCs are composed of ~ 500 copies of nucleoporins, where nucleoporin proteins are arranged in distinct subcomplexes and typically categorized as: cytoplasmic filament, central channel, nuclear basket, transmembrane, and structural (inner & outer ring) nucleoporins [64]. The organizational principles of the NPC are evolutionarily conserved albeit different sizes depending on the organism (Figure 2) [111].

The scaffold pore structure of the NPC is formed by the structural nucleoporins and are hence the largest category of nucleoporin proteins [29]. The Nup107 outer ring complex in vertebrates is composed of nine subunits (Nup107-Nup133-Nup160-Nup96-Nup85/75-Nup43-Nup37-Sec13-SEH1) and stands as the largest structural subcomplex, spanning both the cytoplasmic and nucleoplasmic rings of the NPC [112]. The Nup93 inner ring complex (Nup93-Nup53-Nup155-Nup188-Nup205) is the second major subcomplex recruited to the assembling NPC [113]. Nup93 itself is necessary for the structural aspects of pore formation and, as part of the inner ring complex, harbors the central transport channel. Small ions and molecules (<40kDa) passively shuttle through the NPC, whereas shuttling of larger macromolecules depends on an active, carrier-based transport system that requires signal recognition by transport receptors known as karyopherins (*e.g*. exportins, importins) [63,110]. These larger nuclear transport receptor-cargo bound complexes shuttle rapidly between the nucleus and cytoplasm, relying on their affinity with phenylalanine-glycine rich nucleoporins (FG-Nups) and on a RanGTP-GDP gradient to maintain directionality [63]. This size-dependent restriction is attributed to the narrow cylindrical structure of the NPC central channel, with protruding cytoplasmic filaments and nuclear baskets that allow for docking of cargo before channel transport. Moreover, the presence of dynamic FG-Nups throughout the central channel serves as additional filter to further limit the movement of macromolecules [65–67]. FG-Nups in the central channel hence create a sieve-like mesh through their weak hydrophobic interactions to function as a restrictive gate and thus define the selective permeability of the NPC [68]. While NPCs remain the predominant route for molecular trafficking, it is important to recognize that alternative pathways for nucleocytoplasmic transport exist. Initially observed in the nuclear egress of herpesvirus, several groups report large ribonucleoprotein complexes to transverse the NE through a budding mechanism. By circumventing the NPC, NE budding systems are speculated to be involved in specialized cellular processes, such as protein quality control to impact physiological processes such as mitochondrial integrity and neuromuscular junction maturation [69,114]. Relative to NPC-mediated trafficking, the underlying mechanisms driving NE budding remain to be fully detailed given their potential influence in pathophysiological processes.

Recent studies have shown a close association between nucleoporins, LINC complex proteins, and nuclear lamina fibers for the structural organization of NPCs [115,116]. Targeted depletion of lamin proteins in lower-level organisms (*i.e. C. elegans*) led to abnormal clustering of NPCs toward one side of the nucleus, suggesting that an intact lamina meshwork is necessary for proper NPC distribution (Table 1) [52]. Furthermore, both Lamin A and Lamin B1 were found to directly interact with Nup153, an essential nuclear basket protein involved in NPC anchoring and nuclear protein import [50]. Interestingly, Lamin A mutations in fibroblasts isolated from laminopathy patients exhibit selective disruption in Nup153 binding to both A-type and B-type lamins, resulting in impaired Nup153 localization to the NE and elevated Nup153 enrichment in the nucleoplasm [50]. Similarly, SUN1, but not SUN2, deficiency in primary fibroblasts and HeLa cells altered nuclear morphology and induced similar NPC aggregation at random clustering in the nuclear membrane [85]. SUN1 has also been implicated in NPC assembly during interphase through direct interactions with the NPC transmembrane protein POM121, indicating a need for SUN1 during the early steps of NPC formation [117].

Growing evidence also highlights a pathological relationship between impaired nucleocytoplasmic transport and natural aging [118]. NPCs are poorly maintained with age in post-mitotic cells, as several groups report NPC loss and consequent nuclear transport defects with age, including ECs [119–121]. Much of this is attributed to the lack of NPC turnover in post-mitotic cells and increased susceptibility to damage over time [120,122]. Aging also impairs nucleocytoplasmic transport via disruption of non-nucleoporin proteins, such as nuclear import receptors. For example, old neurons exhibit decreased nuclear import receptor Ran-Binding Protein 17 (RanBP17) and defects in nucleocytoplasmic compartmentalization [123]. Likewise, the nuclear import protein Transportin-1 (TNPO1, also karyopherin-β2) was shown to improperly sequester to the cytoplasm in HGPS fibroblasts, thus affecting TNPO1 cargo proteins [124]. Rather, TNPO1-dependent cargo (*i.e*. Nup153) is not properly imported into the nucleus. This leads to the diminished recruitment of other nucleoporins, resulting in abnormal NPC assembly, chromatin disorganization, and premature entry into cellular senescence.

### Nucleoporins in cardiovascular disease

Recent reports associate several human diseases with perturbed nuclear-cytosolic segregation [125,126]. In quiescent cells, it is well-established that NPCs do not turn over and instead remain incorporated at the nuclear membrane during the entire lifespan of a cell^118,120^. The inability to replace damaged NPCs thus leads to NPC deterioration and increased nuclear permeability over time [120,127]. The adult endothelium is constantly subjected to various stressors and thus particularly vulnerable to aging. It is therefore possible that endothelial NPCs accumulate damage over time, causing premature vascular aging and consequent age-associated CVD. As such, emerging translational work has focused on the role of the NPC and its component nucleoporin proteins as potential mediators for CVDs [119].

Recent clinical studies highlight the substantial changes in protein levels of specific nucleoporins – Nup93 (inner ring), Nup153 (nuclear basket), Nup160 (outer ring), NDC1 (transmembrane domain) – in myocardial cells isolated from cardiomyopathy patients when compared to those of healthy donors (Table 1) [128]. These alterations in nucleoporin levels directly correlate with worsened left ventricular function in both dilated and ischemic patients. In addition, the expression of Nup62, an FG-Nup necessary for size-dependent transport of biomolecules, is markedly reduced in myocardial cells isolated from cardiomyopathy patients and murine models of heart failure [129]. Cardiomyocytes isolated from heart failure patients also exhibit increased nuclei size and impaired import and export function [129]. Similarly, targeted loss of the inner ring protein Nup155 altered nuclear membrane permeability and inhibited the nuclear import of heat shock protein 70 (Hsp70), thereby reducing atrial action potential duration in cardiomyocytes [130]. This is the first report associating nucleoporin abnormalities with heart failure and identifies a novel non-ion channel-based determinant in human atrial fibrillation.

EC health is increasingly recognized as a major factor in preventing age-related vascular disease. Although NPCs serve as the major gateway of molecular transport across the NE, the contributions of NPC proteins in EC and vascular health, unlike those of the LINC complex or nuclear lamina components, remain elusive. An optimal state of EC health relies on the proper subcellular localization of transcription factors to maintain a quiescent and anti-inflammatory gene signature. Alluding to the importance of endothelial nucleoporins, the plant-based *R*-(-)-β-O-methylsynephrine was found to exhibit anti-angiogenic properties via binding to Nup153 and inhibiting its RNA-binding capacity [131]. Targeted knockdown of Nup153 similarly impaired VEGF-induced angiogenesis and subsequent cell viability in both cultured human ECs and transgenic models (Table 1) [131]. It remains unclear, however, the impact of Nup153 loss on endothelial nuclei permeability. Recent studies suggest that cellular senescence, including the endothelium, reflects a state of impaired nucleocytoplasmic transport [119,120,132], leading to the nuclear accumulation of pro-inflammatory transcriptional regulators (*i.e*. Yap, NF-κB, MRTF-A), which cause cellular dysfunction (Figure 1b) [47,133]. Our group recently identified Nup93 as a novel regulator of EC health; endothelial Nup93 expression is substantially reduced in the coronary vasculature of aged mice [119]. Intriguingly, endothelial loss of Nup93 resulted in nuclear pore defects for increased nuclear Yap accumulation and downstream EC inflammation, where inhibiting Yap activity in Nup93-deficient ECs reversed the senescence-associated inflammatory phenotypes [119]. The translational implications of endothelial Nup93 loss, however, will require further investigation using appropriate *in vivo* models.

### Perspectives and future directions

From a cellular perspective, the nuclear membrane is enriched in various structurally influential proteins implicated in nuclear shape and stiffening. The loss of several nuclear membrane proteins (*e.g*. LaminB1, Nup93) has been associated with distorted nuclear morphology for both natural and pathological aging. This recent focus has advanced our appreciation and understanding for NE components as novel and dynamic sensors involved in biophysical force transmission and mechanical signal regulation. While aberrant expression of NE-associated proteins has been shown to alter nuclei integrity and invariably lead to cellular and vessel dysfunction, the contribution of nucleoporin dysregulation in disease is far less understood.

Nucleocytoplasmic exchange of biomolecules is undeniably one of the most essential biological gating mechanisms reliant on various transport features for cellular communication and regulation. As the predominant gateway for molecular trafficking, NPCs have historically been perceived as static structures with the sole purpose of nucleocytoplasmic exchange. Recent studies nonetheless indicate that nucleoporins can also regulate transport-independent features, such as plasma membrane anchoring and mechanobiology [134]. The regulatory capacity of nucleoporins beyond nucleocytoplasmic transport is further illustrated with the discovery that nucleoporins interact with histone modifiers and chromatin-remodeling proteins [111,135,136]. Years of work have uncovered distinct nucleoporins with major roles in epigenetic-associated processes to influence processes such as chromatin architecture [137], transcriptional activation/repression [138], and transcriptional memory [139]. These mechanisms allow cells to rapidly alter gene profiles in response to environmental changes, where epigenetic modifications are well-associated with CVD. Several genes implicated in CVD undergo epigenetic changes to suggest a causal association between epigenetic alterations and the incidence of CVD [140,141]. Nucleoporin perturbations may hence impact the chromatin landscaping of vascular cells to engage epigenomic regulatory mechanisms. Given the selective nature of nucleoporins in tissue-specific chromatin binding patterns, it is particularly interesting to postulate changes in the gene signature of vascular cells with age and/or disease onset, thus presenting a unique opportunity to leverage the power of sequencing technology. Such approaches would reveal NPCs as mediators of vessel chromatin dynamics and deepen our understanding of how NE components can be therapeutically targeted to modulate whole-cell behavior. Nevertheless, nucleoporin dysregulation may serve as an initiating event for EC dysfunction, pointing to its capacity at genetic control via both transport-dependent and transport-independent mechanisms. The discovery of tissue-specific NPC components and differential nucleoporin expression levels across cell and tissue types has garnered the idea that cells may use specialized NPCs for distinct cellular processes [142]. Targeted *in vivo* manipulation of nucleoporin expression would provide insight toward key processes from a cell-specific perspective. Understanding these translational implications will, however, require the generation of novel *in vivo* models to fully understand how NPC disruption affects CV homeostasis and disease. Murine models continue to stand as the gold-standard for modeling human processes but are often limited due to breeding time requirements and cost. Lower-model organisms, such as zebrafish, offer several advantages that have driven their adoption into vascular research. Aside from their rapid development, the mechanisms underlying zebrafish vessel development are often conserved in mammalian vertebrate models to inform our understanding of human vascular pathogenesis [143,144]. Furthermore, the optical clarity of zebrafish has facilitated the integration of numerous fluorescent transgenic lines for high-resolution imaging, thus allowing for the visualization of vascular structures, lineage tracing studies, and cell-type specific labeling – approaches that are often unfeasible in murine models. Lastly, the nuclear pore field is equipped with various cutting-edge cell biology techniques to assess NPC structure and nuclear transport properties. As such, the nuclear envelope field offers a rich knowledge base as we begin to comprehensively analyze how nuclear properties influence vascular cells and CVD. The integration of such resources offers exciting opportunities for cardiovascular researchers, where these initial studies are beginning to highlight a major knowledge gap with tremendous therapeutic potential.

## Data Availability

Data sharing is not applicable to this article as no new data was created or analyzed in this study.
